# The Effects of Dietary *Enterococcus faecalis* HHP003 Supplementation on Gut Microbiota Composition and Gut Health in Cats with Mild Diarrhea

**DOI:** 10.3390/ani16091366

**Published:** 2026-04-29

**Authors:** Qin Wang, Yanyi Zheng, Wenyu Huang, Feitong Liu, Lingling Zhao, Siyuan Xue, Huiwen Zeng, Yi Wu

**Affiliations:** 1 State Key Laboratory of Animal Nutrition and Feeding, College of Animal Science and Technology, China Agricultural University, Beijing 100193, China; wangqin2025@cau.edu.cn (Q.W.); 2022304010206@cau.edu.cn (S.X.); zenghuiwen.phd@hotmail.com (H.Z.); 2 H&H Group, H&H Research, China Research and Innovation Center, Guangzhou 510700, China; ali.zheng@hh.global (Y.Z.); riley.huang@hh.global (W.H.); erin.liu@hh.global (F.L.); lynn.zhao@hh.global (L.Z.)

**Keywords:** cats, *Enterococcus faecalis* HHP003, gut microbiota, intestinal inflammation, metabolites

## Abstract

As a probiotic lactic acid bacterium, *E. faecalis* regulates intestinal flora, strengthens the intestinal barrier, and enhances immunity in the host. However, as a new strain isolated from healthy feline gut, *E. faecalis* HHP003 has an unclear effect on feline intestinal health. Twenty cats with mild diarrhea were randomly assigned to two groups: one group received the standard diet (MD), while the other group received supplemental *E. faecalis* HHP003 (EF). Meanwhile, 10 healthy cats were enrolled as a healthy control group (CON). After 42 days of intervention, the EF group exhibited significantly reduced serum inflammatory markers (TNF-α and IL-1β), as well as decreased levels of calprotectin and lipopolysaccharide, compared with the MD group (*p* < 0.05). The intestinal microbial diversity was altered in the EF group, with increased Chao and Shannon indices (*p* < 0.05). Specifically, the relative abundances of Bacillota, Bacteroidota, and Ruminococcaceae were significantly higher in the EF group than those in the MD group (*p* < 0.05). Metabolomic analysis identified 697 differential metabolites and nine KEGG metabolic pathways (*p* < 0.05) between the EF and MD groups. Furthermore, the study identified significant associations between the gut microbiota and selected serum metabolites. In summary, *E. faecalis* HHP003 supplementation was associated with reduced serum inflammatory responses, improved intestinal inflammation and barrier markers, and altered gut microbiota and serum metabolite levels in cats with mild diarrhea.

## Simple Summary

Diet is a key factor in regulating feline gut health, and probiotics are promising functional supplements for pet foods to maintain gastrointestinal homeostasis. The impact of *Enterococcus faecalis* (*E. faecalis*) HHP003 on intestinal health in cats experiencing mild diarrhea remains underexplored. This study showed that supplementing cats’ diets with *E. faecalis* HHP003 enhanced feline intestinal health by altering the gut microbiota and blood metabolites, offering an effective strategy for supporting feline gastrointestinal function.

## 1. Introduction

As living standards continue to rise, pets have become an increasingly integral part of daily life for many families. In response, a growing number of owners are placing greater emphasis on the health of their feline companions. Among various aspects of well-being, intestinal health stands out as a fundamental component, directly influencing nutrient digestion, absorption, and immune competence [1]. Beyond its contribution to assimilating nutrients and breaking down food, the mammalian intestine also functions as the largest immune organ in the body [2]. Its condition is thus widely regarded as a cornerstone of systemic health in the host [3]. Maintaining this intestinal equilibrium, however, is a multifaceted process that depends not only on the integrity of the host’s physical barrier but also on intricate interactions with the dense and diverse gut microbial community [4]. In this context, the gut microbiota influences host physiology through metabolic activities, immune regulation, and the exclusion of pathogenic microorganisms, and is often described as a vital “metabolic organ” [5].

The Bacteroidetes, Proteobacteria, Actinobacteria, and Firmicutes phyla constitute the primary bacterial phyla in the feline gut microbiota, whose relative abundances are shaped by a range of factors including genetic background, age, dietary habits, housing conditions, and physiological status [6]. Given that gastrointestinal health directly influences growth performance, reproductive efficiency, and longevity in cats, disruptions to this microbial ecosystem have been implicated in the pathogenesis of several common digestive disorders, such as diarrhea, constipation, and feline inflammatory bowel disease (IBD). These conditions are typically characterized by shifts in dominant bacterial abundance, an overgrowth of pathogens, and a reduction in beneficial microbes [7,8]. Employing fluorescence in situ hybridization to compare colonic microbiota between healthy cats and those diagnosed with feline IBD, one investigation revealed that populations of *Bifidobacterium* and *Bacteroides* were notably reduced in diseased animals, while sulfide-producing *Desulfovibrio* species were significantly enriched—findings that reinforce the intricate link between IBD and microbial imbalance [9]. Similarly, a systematic investigation into the intestinal microbial composition of felines with chronic IBD further elucidated the association between disease and gut microbiota dysregulation, drawing upon clinical, microbiological, and histopathological lines of evidence [10].

*Enterococcus faecalis* (*E. faecalis*) is known for its considerable adaptability to diverse environmental conditions. As a prevalent commensal organism, it naturally inhabits the gastrointestinal tract, oral cavity, and reproductive system of both animals and humans, while also being widely distributed within various natural ecosystems [11]. Owing to its facultative anaerobic nature, this bacterium possesses both aerobic and anaerobic metabolic pathways, which contribute to its robustness and ability to withstand environmental stressors [12]. Accumulating evidence suggests that *E. faecalis* not only displays antimicrobial activity and probiotic attributes, but also contributes to improved host performance and the stabilization of microbial communities, positioning it as a key species in the maintenance of intestinal equilibrium [13,14]. Classified within the lactic acid bacteria group, it is extensively utilized in the food sector for applications including fermentation processes, flavor development, and the production of bacteriocins [15]. The incorporation of probiotics into feline diets has been shown to modulate immune responses, mitigate stress effects, suppress pathogenic organisms, and improve growth metrics [16]. As a probiotic increasingly employed in animal husbandry, *E. faecalis* has demonstrated efficacy in enhancing growth rates and gastrointestinal health while reducing the incidence of diarrhea. For instance, piglets receiving *E. faecalis* supplementation exhibited significantly elevated α-diversity and species richness in their fecal microbiota relative to control groups [17]. Similarly, research has indicated that *E. faecalis* can optimize feed conversion ratios and promote intestinal well-being in broiler chickens [18]. Despite these findings in other species, investigations into the effects of dietary *E. faecalis* supplementation on the intestinal health of felines remain relatively scarce.

Therefore, the present study aimed to investigate the effects of dietary *E. faecalis* HHP003 supplementation on the gut microbiota composition, inflammatory response, and intestinal barrier in cats with mild diarrhea.

## 2. Materials and Methods

### 2.1. Animals

Experimental protocols of animal handling and dietary treatments were reviewed and approved by the Institutional Animal Care and Use Committee of China Agricultural University (No. AW41805202-1-1). A total of 30 intact adult cats aged 1 to 7 years were used in this study. Breeds included Ragdoll, Maine Coon, British Shorthair, American Shorthair, Golden Gradient, and Domestic Shorthair. Among them, 20 cats had mild diarrhea and 10 were clinically healthy. Cats with mild diarrhea had a fecal score of 3.5–4.5, whereas healthy cats had a fecal score of 2.5–3.5 [19]. All cats had a body condition score of 4–5 [20]. Cats with mild diarrhea had no other clinical signs except for diarrhea. No animals included in this study had a history of allergies or immune-mediated diseases, parasitic infestations, hepatic disease, renal disease, or pancreatic insufficiency. None of these cats had been administered medications, dietary interventions, antibiotics, or immunosuppressive drugs, nor had they undergone surgery within three months prior to the study. Cats in pregnancy or lactation, or those unable to eat or be fed orally, were excluded from the experiment.

During the experiment, all cats were individually housed in separate cages measuring 1.5 m × 1.5 m × 2.0 m, and an appropriate temperature (23–26 °C) and humidity (50–60%) were maintained. The animal rooms were kept ventilated and clean through daily cleaning and routine disinfection in accordance with standard sanitation protocols. During the study, cats could freely obtain water and food and were closely monitored for behavior, appetite, and fecal consistency.

### 2.2. Experimental Design and Sample Collection

The study comprised 20 adult cats with mild diarrhea randomly divided into two groups (n = 10, half females and half males) based on body weight and fecal score. Feeding procedures are described below: (1) mild diarrhea group (MD): cats with mild diarrhea were fed a basal diet; (2) experimental group (EF): cats with mild diarrhea were fed a basal diet and supplemented with 6 × 10^10^ CFU/kg diet of *E. faecalis* HHP003. Meanwhile, 10 healthy cats fed a basal diet were included in the control group (CON). The basal diet formulation used in this study meets the nutritional requirements recommended by the NRC (2006) (Table 1). *E. faecalis* was certified as a feed additive by the Ministry of Agriculture of China. *E. faecalis* HHP003 (A-A-2) was isolated from the feces of healthy cats, preserved in the Guangdong Microbial Culture Collection Center (GDMCC, Deposit No. 62869), and provided by H&H Group (Guangzhou), China. The safety of *E. faecalis* HHP003 was evaluated by an independent third-party testing authority, the China Center of Industrial Culture Collection (CICC), which is accredited with CNAS (China National Accreditation Service for Conformity Assessment, Accreditation No. L9421), CMA (China Inspection Body and Laboratory Mandatory Approval, No. 240000349907), and ILAC MRA (International Laboratory Accreditation Cooperation Mutual Recognition Arrangement) certifications. Standardized acute pathogenicity tests were performed in SPF ICR mice according to *GB 31615.2-2025* National Food Safety Standard: Safety Assessment Procedure for Microbial Starters Used in Food [21], including both oral and intraperitoneal administration routes. The test was conducted in accordance with good laboratory practice (GLP), and the official test report (No. 25-1047-02035.03-04258) was used as the primary safety evidence. Body weight and fecal score (scoring criteria: Appendix A Table A1) were measured weekly in all groups to assess growth performance and fecal characteristics, respectively. Fecal scoring was performed by an investigator blinded to the group allocation. Additionally, fresh fecal samples collected on days 28 and 42 were used for enzyme-linked immunosorbent assay (ELISA) analyses of calprotectin and CRP.

On days 28 and 42 of the experiment, we collected blood samples from the limb veins after 12 h of fasting and placed them into vacuum blood collection tubes without anticoagulant. These samples were then centrifuged at 3000 rpm at 4 °C for 10 min to obtain serum for ELISA analyses, and the serum collected on day 42 was subjected to untargeted metabolomic analysis. On day 42, fecal material was gathered immediately from all felines after defecation, snap-frozen in liquid nitrogen, and stored at −80 °C for microbial diversity and abundance analysis.

### 2.3. Sample Analysis

#### 2.3.1. Detection of Cytokines, Intestinal Inflammation, and Barrier Biomarkers

The serum concentrations of inflammatory factors including interleukin-1β (IL-1β), IL-6 and tumor necrosis factor-α (TNF-α) were analyzed using commercially available ELISA kits following the manufacturer’s instructions. Fecal calprotectin and fecal C-reactive protein (CRP) levels as key biomarkers for evaluating intestinal inflammation were also measured using ELISA kits. The levels of lipopolysaccharide (LPS) and zonulin were quantified using commercial ELISA kits. All commercial kits used in this study were purchased from Jiangsu Meimian Industrial Co., Ltd. (Yancheng, China). Detailed detection information for the ELISA kits is presented in Appendix A Table A2.

#### 2.3.2. Fecal Microbiota Analysis

Fecal total bacterial DNA was extracted using the QIAamp Fast DNA Stool Mini Kit (Qiagen, Hilden, Germany). Amplification of 16S rRNA from the V3–V4 regions was performed using primers 341F (5′-CCTAYGGGRBGCASCAG-3′) and 806R (5′-GGACTACHVGGGTWTCTAAT-3′). Equimolar quantities of purified amplicons were combined and sequenced on the Illumina MiSeq platform using 2 × 300 bp paired-end chemistry (Illumina, San Diego, CA, USA). Raw sequencing data underwent quality trimming with fastp (v0.19.6), and then read merging using FLASH (v1.2.11). High-quality reads were clustered into operational taxonomic units (OTUs) at a 97% identity cutoff using the UPARSE pipeline (v11). Taxonomic assignment of OTU representatives was performed with the RDP classifier (v2.2) referencing the Silva 138 database at a 0.7 confidence threshold. Community richness and diversity indices were generated using Mothur (v1.30.2). Between-sample dissimilarities were quantified using β-Curtis metrics and visualized through principal co-ordinate analysis (PCoA). Taxon-specific community profiles were constructed across all phylogenetic levels. Statistical comparisons of microbial abundances between experimental groups were conducted using the Kruskal–Wallis H test.

#### 2.3.3. Untargeted Metabolomics Analysis

An amount of 100 μL of serum was added to a 1.5 mL centrifuge tube, followed by 400 μL of extraction solution (acetonitrile:methanol = 1:1, volume ratio) containing four internal standards. After vortex mixing for 30 s, low-temperature ultrasonic extraction was performed for 30 min at 5 °C and 40 KHz, and then the samples were stored at −20 °C for 30 min. Subsequently, samples were centrifuged at 13,000× *g* and 4 °C for 15 min, and the supernatants were transferred and dried with nitrogen. The residue was reconstituted with 100 μL of reconstitution solution (acetonitrile:methanol = 1:1, *v*/*v*), followed by low-temperature ultrasonic extraction at 5 °C and 40KHz for 5 min. The samples were then centrifuged at 13,000× *g* and 4 °C for 10 min, and the resulting supernatant was carefully transferred to an injection vial equipped with a micro-insert for instrumental analysis.

For the untargeted metabolomics analysis, an ACQUITY HSS T3 chromatographic column (Waters Corporation, Milford, MA, USA) and a UHPLC-Orbitrap Exploris 240 system (Thermo Fisher Scientific, Waltham, MA, USA) were used. Raw data obtained from liquid chromatography–mass spectrometry (LC-MS) were analyzed and processed using Progenesis QI (v2.3) software (Waters Corporation, Milford, MA, USA), including peak detection, peak alignment, retention time correction integration, and baseline filtering. The final output comprised a data matrix containing the mass-to-charge ratio (*m*/*z*), peak intensity, and retention time. Simultaneously, spectral information from secondary mass spectrometry (MS/MS) and mass spectrometry (MS) was matched against Metlin (https://metlin.scripps.edu/), the public metabolomics database HMDB (https://www.hmdb.ca/), and Majorbio internal database to obtain metabolite information. The database-searched data matrix was uploaded to the Majorbio Cloud Platform (cloud.majorbio.com) for further analysis.

### 2.4. Statistical Analysis and Visualization

We used GraphPad Prism 9.0.0 (San Diego, CA, USA) and IBM SPSS Statistics 27.0 (Chicago, IL, USA) to analyze the data. Statistical significance of the differences between groups was determined by two-way repeated measures ANOVA, and multiple comparisons were adjusted using the Benjamini–Hochberg (FDR-adjusted *p* < 0.05) method. The identification of significantly differential metabolites in untargeted metabolomics was based on a VIP > 1 from the OPLS-DA model and a *p* < 0.05 by Student’s *t*-test. Visualization of microbial sequencing data was performed using R software (v4.0). Heatmaps were generated using the R vegan package (v2.6-4), and bacterial community bar charts were plotted using the R ggplot package (v3.3.1). Relative abundance of microbial communities was assessed using the Kruskal–Wallis H test. Statistical significance was set at *p* < 0.05, where 0.05 ≤ *p* < 0.1 indicates a significant trend, and *p* < 0.001 indicates a highly significant difference.

## 3. Results

### 3.1. Physical Characteristics

**Figure 1:**
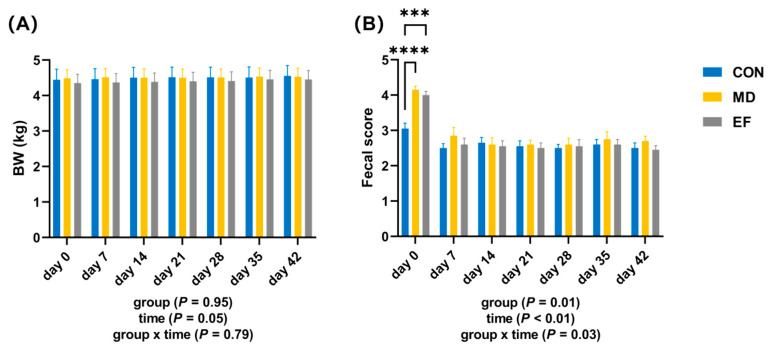
Effects of *E. faecalis* HHP003 on body weight and fecal score in cats. (**A**) Body weight on days 0, 7, 14, 21, 28, 35, and 42. (**B**) Fecal score were recorded on days 0, 7, 14, 21, 28, 35, and 42. CON, healthy cats receive a basal diet; MD, cats with mild diarrhea receive a basal diet; EF, cats with mild diarrhea receive a basal diet blended with 6 × 10^10^ CFU/kg of *E. faecalis* HHP003. BW, body weight. Express values as means ± SEMs, n = 10. Different letters on the bar chart indicate significant differences between groups (*p* < 0.05). * *p* < 0.05, ** *p* < 0.01, *** *p* < 0.001, and **** *p* < 0.0001.

Throughout the experimental period, no significant difference in body weight (BW) was detected among the three groups (*p* ≥ 0.05; Figure 1A). On day 0, fecal scores in the MD and EF groups were significantly higher than those in the CON group (*p* < 0.001; Figure 1B). However, on days 7, 14, 21, 28, 35, and 42, no significant differences in fecal scores were observed among the three groups (*p* ≥ 0.05; Figure 1B). The fecal scores on day 0 were significantly higher in both the MD and EF groups compared to other time points (*p* < 0.05; Figure 1B).

### 3.2. Serum Inflammatory Cytokines

**Figure 2:**
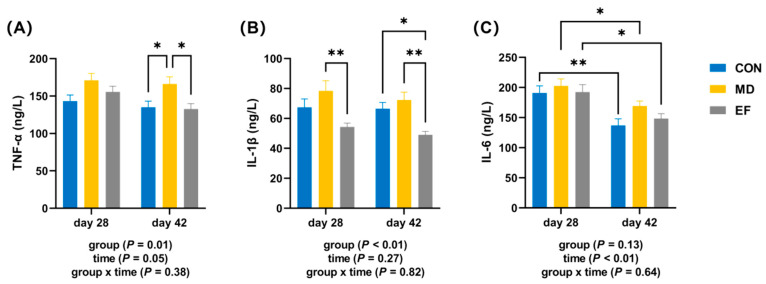
The effects of *E. faecalis* HHP003 on the concentrations of serum inflammatory cytokines in cats. (**A**) Tumor necrosis factor (TNF-α) levels. (**B**) Interleukin (IL)- β levels. (**C**) IL-6 levels. CON, healthy cats receive a basal diet; MD, cats with mild diarrhea receive a basal diet; EF, cats with mild diarrhea receive a basal diet blended with 6 × 10^10^ CFU/kg of *E. faecalis* HHP003. Express values as means ± SEMs, n = 10. * *p* < 0.05, ** *p* < 0.01, *** *p* < 0.001, and **** *p* < 0.0001.

On day 42 of the experiment, compared with the MD group, the serum TNF-α levels in cats from not only the CON group but also the EF group were significantly lower (*p* < 0.05; Figure 2A). Meanwhile, on days 28 and 42, the serum IL-1β level in the EF group was significantly lower than that in the MD group (*p* < 0.05; Figure 2B). Moreover, on day 42, a markedly lower serum IL-1β level was detected in the EF group relative to the CON group (*p* < 0.05; Figure 2B). In contrast, no appreciable differences in serum IL-6 concentrations were observed among all three groups at either day 28 or day 42 (*p* ≥ 0.05; Figure 2C). However, the IL-6 levels in all three groups were significantly lower on day 28 than on day 42 (*p* < 0.05; Figure 2C).

### 3.3. Intestinal Inflammation and Barrier Function Parameters

**Figure 3:**
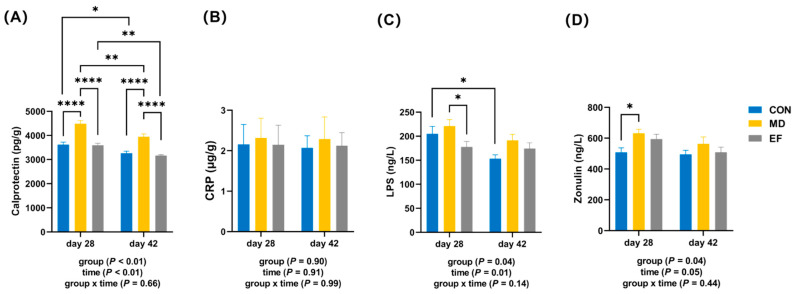
The effects of *E. faecalis* HHP003 on intestinal inflammation and barrier function parameters in cats. (**A**) Fecal calprotectin levels. (**B**) Fecal C-reactive protein (CRP) levels. (**C**) Lipopolysaccharide (LPS) levels. (**D**) Zonulin levels. CON, healthy cats receive a basal diet; MD, cats with mild diarrhea receive a basal diet; EF, cats with mild diarrhea receive a basal diet blended with 6 × 10^10^ CFU/kg of *E. faecalis* HHP003. Express values as means ± SEMs, n = 10. * *p* < 0.05, ** *p* < 0.01, *** *p* < 0.001, and **** *p* < 0.0001.

On days 28 and 42 of the experiment, compared with the MD group, the fecal calprotectin levels in cats from both the CON and EF groups were significantly lower (*p* < 0.05; Figure 3A). However, the CRP level showed no significant differences among the three groups at different time points (*p* ≥ 0.05; Figure 3B). Notably, on day 28, the LPS level was significantly decreased in the EF group compared with the MD group (*p* < 0.05; Figure 3C) and the zonulin level in the CON group was significantly lower than that in the MD group (*p* < 0.05; Figure 3D). However, the calprotectin levels in all three groups were significantly lower on day 28 than on day 42 (*p* < 0.05; Figure 3A). The LPS level in the CON group on day 28 was significantly lower than that on day 42 (*p* < 0.05; Figure 3C).

### 3.4. Fecal Metagenomic Profiling of Gut Microbiota

**Figure 4:**
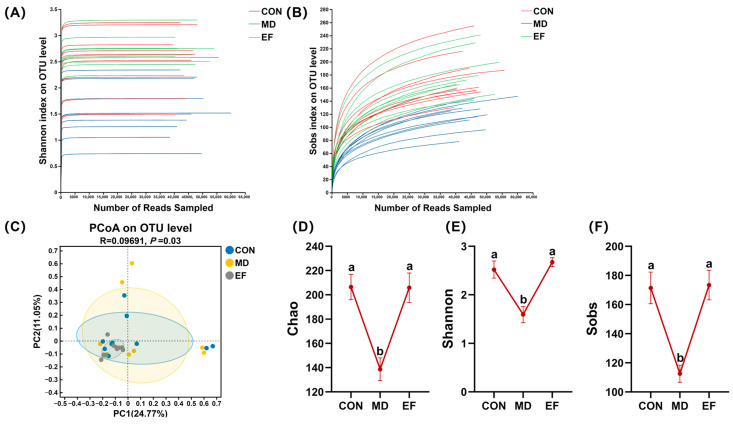
The fecal microbiota β-diversity and α-diversity analysis. (**A**,**B**) Rarefaction curves based on the Shannon and Sobs indices. (**C**) Principal co-ordinates analysis (PCoA) based on OTU level composition. (**D**–**F**) Assessment of α-diversity via the Chao, Shannon, and Sobs indices. CON, healthy cats receive a basal diet; MD, cats with mild diarrhea receive a basal diet; EF, cats with mild diarrhea receive a basal diet blended with 6 × 10^10^ CFU/kg of *E. faecalis* HHP003. Express values as means ± SEMs, n = 10. Different letters indicate significant differences (*p* < 0.05).

The rarefaction curves based on the Shannon (Figure 4A) and Sobs (Figure 4B) indices showed that all curves gradually approached a plateau as the sequencing depth increased, indicating that the sequencing depth was sufficient to capture the majority of microbial diversity in all samples. PCoA based on OTU-level data demonstrated a clear clustering separation among the fecal microbial communities of the three experimental groups (*p* < 0.05; Figure 4C). Additionally, α-diversity analysis revealed that the Chao index was significantly elevated in the CON and EF groups, while the Shannon and Sobs indices also showed a marked increase, relative to the MD group (*p* < 0.05; Figure 4D–F).

**Figure 5:**
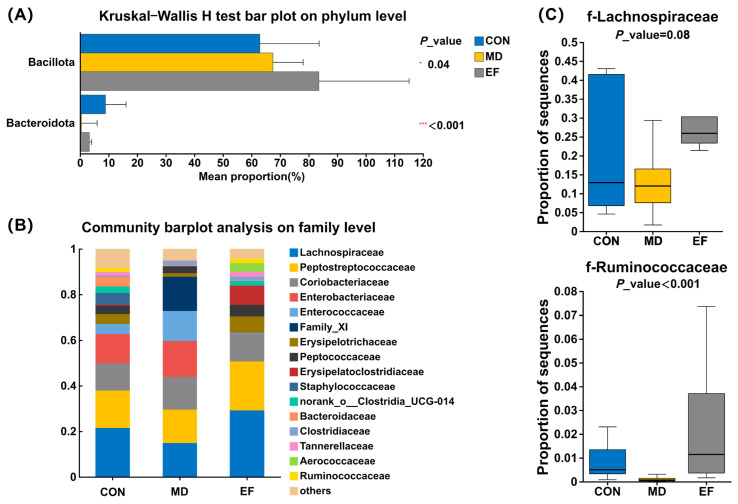
The relative abundance of bacterial communities at the phylum level (**A**) and family (**B**) level on day 42. (**C**) The abundance of Lachnospiraceae and Ruminococcaceae. CON, healthy cats receive a basal diet; MD, cats with mild diarrhea receive a basal diet; EF, cats with mild diarrhea receive a basal diet blended with 6 × 10^10^ CFU/kg of *E. faecalis* HHP003. n = 10. * *p* < 0.05, ** *p* < 0.01, *** *p* < 0.001, and **** *p* < 0.0001.

Fecal microbial abundance was profiled according to phylum and family classifications (Figure 5). At the phylum level (Figure 5A), the relative abundance of Bacillota in the feces of cats in the EF group was significantly higher than that in the CON group (*p* < 0.05). In addition, compared with the CON group, the relative abundance of Bacteroidota in the MD group was significantly decreased (*p* < 0.05), while there was no significant change in the EF group (*p* ≥ 0.05). At the family level (Figure 5B), Lachnospiraceae (CON: 21.47%; MD: 14.86%; EF: 29.16%), Peptostreptococcaceae (CON: 16.38%; MD: 14.66%; EF: 21.57%), Coriobacteriaceae (CON: 12.00%; MD: 14.43%; EF: 12.06%), Enterobacteriaceae (CON: 12.85%; MD: 15.72%; EF: 0.02%), and Ruminococcaceae (CON: 1.65%; MD: 0.09%; EF: 2.01%) were the predominant families. Compared with the CON and MD groups (Figure 5C), cats in the EF group showed an increasing trend in the abundance of Lachnospiraceae (*p* = 0.08); notably, Ruminococcaceae exhibited considerably higher abundance (*p* < 0.001).

**Figure 6:**
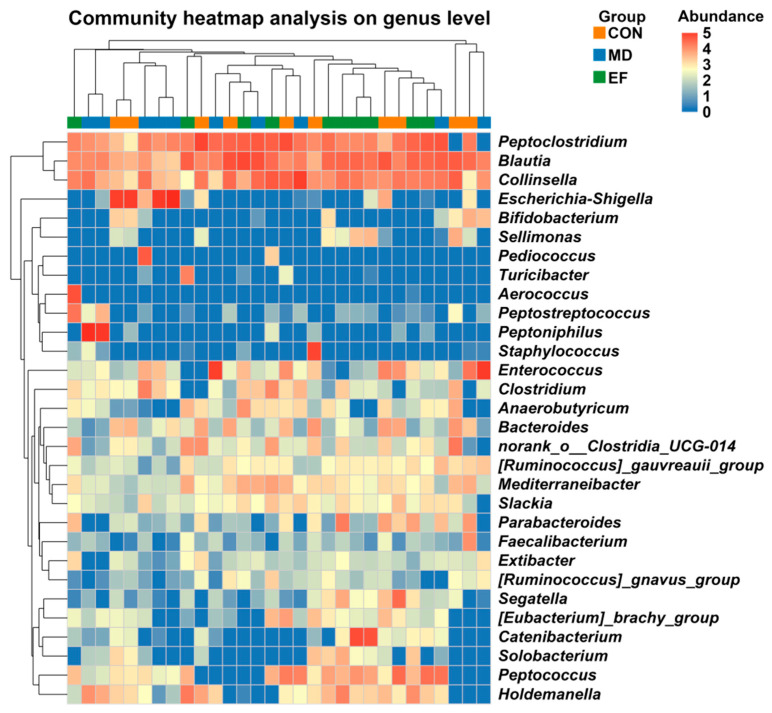
Genus-level relative abundance of bacterial communities at day 42. CON, healthy cats receive a basal diet; MD, cats with mild diarrhea receive a basal diet; EF, cats with mild diarrhea receive a basal diet blended with 6 × 10^10^ CFU/kg of *E. faecalis* HHP003. n = 10.

The relative abundance of fecal bacteria was also measured at the genus levels (Figure 6). *Blautia* (CON: 15.89%; MD: 12.71%; EF: 23.26%), *Peptoclostridium* (CON: 15.93%; MD: 14.26%; EF: 17.75%), *Collinsella* (CON: 12.00%; MD: 14.43%; EF: 12.06%), *Escherichia-Shigella* (CON: 12.85%; MD: 15.71%; EF: 0.02%), and *Enterococcus* (CON: 4.38%; MD: 13.08%; EF: 0.44%) were the predominant genera.

**Figure 7:**
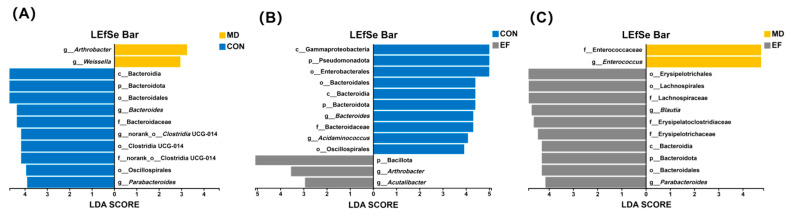
Linear discriminant analysis (LDA) coupled with effect size measurements identifies taxa with significantly different abundances among the groups. Only the top 10 taxa with LDA scores above the threshold of 2 are shown in the figure. (**A**) Differentially abundant bacteria between the MD and CON groups. (**B**) Differentially abundant bacteria between the CON and EF groups. (**C**) Differentially abundant bacteria between the MD and EF groups. CON, healthy cats receive a basal diet; MD, cats with mild diarrhea receive a basal diet; EF, cats with mild diarrhea receive a basal diet blended with 6 × 10^10^ CFU/kg of *E. faecalis* HHP003. n = 10.

The linear discriminant analysis effect size (LEfSe) algorithm was further used to analyze the microbial composition from phylum to genus levels among the groups (Figure 7). Compared with the CON group, *Arthrobacter* and *Weissella* were enriched in the MD group (*p* < 0.05; Figure 7A). Bacillota, *Arthrobacter*, and *Acutalibacter* were enriched in the EF group compared with the CON group (*p* < 0.05; Figure 7B). Lachnospiraceae, *Blautia*, Bacteroidota, and *Parabacteroides* were the dominant enriched taxa in the EF group, whereas Enterococcaceae and *Enterococcus* were enriched in the MD group (*p* < 0.05; Figure 7C).

### 3.5. Untargeted Serum Metabolomics Analysis

**Figure 8:**
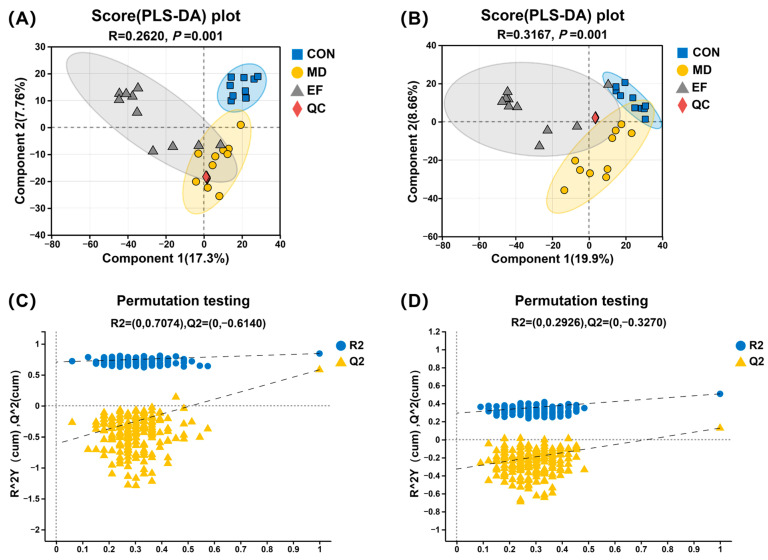
Partial least squares discriminant analysis (PLS-DA) was performed to visualize the metabolic differences among groups. The PLS-DA model was validated using 200 permutation tests to prevent overfitting, with a confidence level of 0.95, and analysis of similarities was used to evaluate the significance of inter-group differences. PLS-DA score plots in positive (**A**) and negative (**B**) ion modes. PLS-DA permutation test plots in positive (**C**) and negative (**D**) ion modes. CON, healthy cats receive a basal diet; MD, cats with mild diarrhea receive a basal diet; EF, cats with mild diarrhea receive a basal diet blended with 6 × 10^10^ CFU/kg of *E. faecalis* HHP003; QC, quality control samples. n = 10.

PLS-DA plots in positive and negative ion modes revealed significant separation of metabolite profiles among the three groups and quality control samples (QC) group (*p* < 0.05; Figure 8A,B). The validation results of the PLS-DA model showed that the R^2^ values in both positive and negative ion modes were higher than the corresponding Q^2^ values. Additionally, the intercepts of the Q^2^ regression lines with the vertical Y-axis were −0.6140 and −0.3270 for the positive and negative ion modes, respectively, suggesting adequate model fitness for subsequent data analysis (Figure 8C,D).

**Figure 9:**
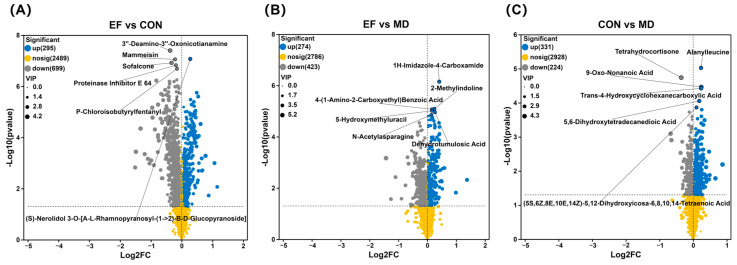
Serum metabolite profiles among the different treatment groups were compared using unpaired Student’s *t*-test (two-tailed). Differential metabolite volcano plots for the comparisons: EF vs. CON (**A**), EF vs. MD (**B**), and CON vs. MD (**C**). CON, healthy cats receive a basal diet; MD, cats with mild diarrhea receive a basal diet; EF, cats with mild diarrhea receive a basal diet blended with 6 × 10^10^ CFU/kg of *E. faecalis* HHP003. Express values as means ± SEMs, n = 10.

Differential metabolites between different groups are shown in Figure 9. Comparative analysis between the EF and CON groups revealed a total of 994 metabolites with significantly altered abundances. Among these, 295 were up-regulated, while 699 were down-regulated in the EF group. Among the top six differential metabolites, (S)-Nerolidol 3-O-[A-L-Rhamnopyranosyl-(1->2)-B-D-Glucopyranoside] was up-regulated (Figure 9A). A total of 697 differential metabolites were identified between the EF and MD groups, with 274 up-regulated and 423 down-regulated. The up-regulated metabolites included 1H-Imidazole-4-Carboxamide, 2-Methylindoline, 4-(1-Amino-2-Carboxyethyl) Benzoic Acid, 5-Hydroxymethyluracil, N-Acetylasparagine, and Dehydrotumulosic acid (Figure 9B). A total of 555 differential metabolites were detected in the CON versus MD comparison, comprising 331 that exhibited significantly increased levels and 224 that showed decreased levels. These up-regulated metabolites included Alanylleucine, 9-Oxo-Nonanoic Acid, Trans-4-Hydroxycyclohexanecarboxylic Acid, 5,6-Dihydroxytetradecanedioic Acid, and (5S,6Z,8E,10E,14Z)-5,12-Dihydroxyicosa-6,8,10,14-Tetraenoic Acid (Figure 9C).

**Figure 10:**
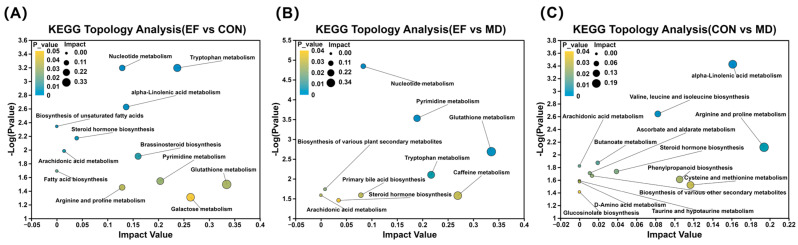
Differential metabolic pathways were identified via KEGG topological analysis using relative-betweenness centrality, with Benjamini–Hochberg multiple testing correction. (**A**) EF vs. CON; (**B**) EF vs. MD; (**C**) CON vs. MD. CON, healthy cats receive a basal diet; MD, cats with mild diarrhea receive a basal diet; EF, cats with mild diarrhea receive a basal diet blended with 6 × 10^10^ CFU/kg of *E. faecalis* HHP003. Express values as means ± SEMs, n = 10.

Pathway topology analysis was conducted based on high-quality KEGG pathways (Figure 10). A total of 12 pathways showed significantly different pathways were identified in the EF group compared with the CON group (*p* < 0.05), namely glutathione metabolism, galactose metabolism, tryptophan metabolism, pyrimidine metabolism, brassinosteroid biosynthesis, alpha-linolenic acid metabolism, nucleotide metabolism, arginine and proline metabolism, steroid hormone biosynthesis, arachidonic acid metabolism, biosynthesis of unsaturated fatty acids, and fatty acid biosynthesis (Figure 10A). Nine pathways, including glutathione metabolism, caffeine metabolism, tryptophan metabolism, pyrimidine metabolism, nucleotide metabolism, primary bile acid biosynthesis, steroid hormone biosynthesis, biosynthesis of various plant secondary metabolites, and arachidonic acid metabolism, showed marked differences in the EF group compared with the MD group (*p* < 0.05; Figure 10B). In addition, 13 metabolic pathways showed significant differences in the CON group compared with the MD group (*p* < 0.05), such as glucosinolate biosynthesis, arachidonic acid metabolism, taurine and hypotaurine metabolism, alpha-linolenic acid metabolism, ascorbate and aldarate metabolism, cysteine and methionine metabolism, valine, leucine and isoleucine biosynthesis, arginine and proline metabolism, butanoate metabolism, steroid hormone biosynthesis, D-amino acid metabolism, phenylpropanoid biosynthesis, and the biosynthesis of various other secondary metabolites (Figure 10C).

### 3.6. Correlation Between Genus-Level Gut Microbiota and Differential Metabolites

**Figure 11:**
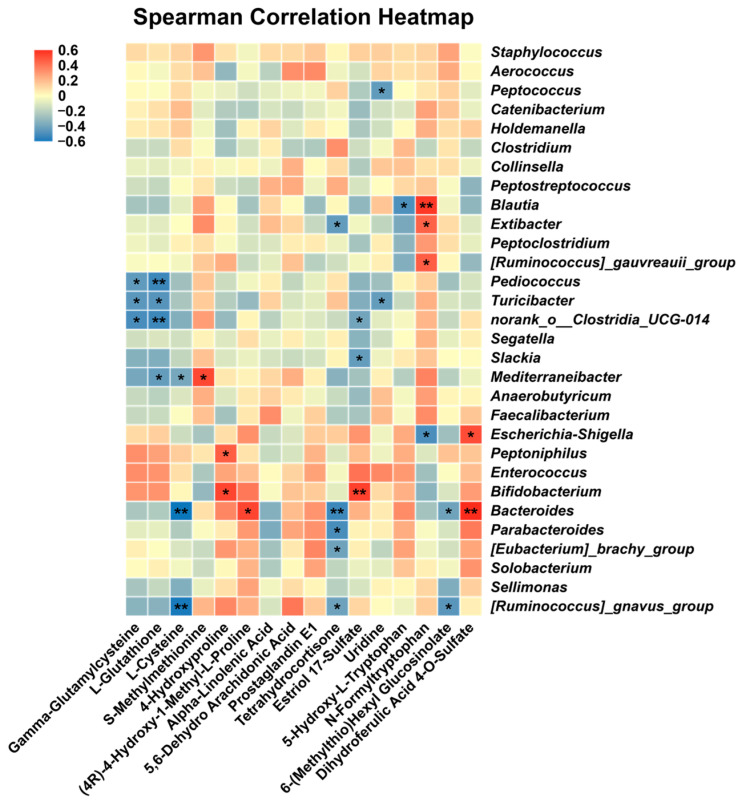
Relationships between screened serum metabolites and genus-level composition of the fecal microbiota. CON, healthy cats receive a basal diet; MD, cats with mild diarrhea receive a basal diet; EF, cats with mild diarrhea receive a basal diet blended with 6 × 10^10^ CFU/kg of *E. faecalis* HHP003. n = 10. * *p* < 0.05, ** *p* < 0.01, *** *p* < 0.001, and **** *p* < 0.0001.

The correlation between serum differential metabolites and genus-level microbiota was assessed using Spearman’s correlation analysis (Figure 11). 4-Hydroxyproline was positively correlated with *Peptoniphilus* and *Bifidobacterium* (*p* < 0.05). Metabolite 5-Hydroxy-L-Tryptophan was negatively correlated with *Blautia* (*p* < 0.05). N-Formyltryptophan metabolite was negatively correlated with *Escherichia-Shigella*, while it was positively correlated with *Blautia*, *Extibacter*, and *[Ruminococcus]_gauvreauii_group* (*p* < 0.05). There was a significant positive correlation between metabolite Dihydroferulic Acid 4-O-Sulfate and *Escherichia-Shigella*, as well as *Bacteroides* (*p* < 0.05).

## 4. Discussion

The gut microbiota plays a key role in maintaining feline health and preventing diseases, and it functions as a critical element in strengthening barrier integrity, elevating immune function, and resisting intestinal pathogens, which are critical for sustaining intestinal homeostasis [22,23]. Intestinal disorders in cats are frequently linked to gut dysbiosis and functional decline, which are featured by reduced microbial diversity and quantity, as well as shifts in microbiota composition and metabolic profiles [4,24]. Probiotics, as a safe and effective intervention, can modulate the gut microbiota and maintain the balance of the intestinal microecosystem [25]. Probiotics based on *E. faecalis* have been identified as effective dietary supplements, which exert beneficial effects on humans and animals by boosting intestinal immune function and modulating the composition of the gut microbiota [11]. However, the effects of *E. faecalis* HHP003 on serum inflammatory status, intestinal barrier integrity, and microbiome composition in cats have not been investigated previously.

Cytokines are a class of endogenous peptides primarily produced by immune cells. They regulate diverse physiological functions and play an indispensable role in modulating various immune responses. Maintaining the balance between pro-inflammatory and anti-inflammatory cytokines is crucial for sustaining normal immune homeostasis and physiological functions [26]. Among these, TNF-α and IL-1β are well-established pro-inflammatory mediators [27]. Meanwhile, TNF-α functions as a crucial mediator involved in intestinal inflammation, and it can promote the secretion of IL-1β by immune cells including macrophages and dendritic cells within the intestinal lamina propria [28]. Studies have found that when piglets challenged with LPS were fed a diet supplemented with *E. faecalis*, the levels of TNF-α and IL-1β in the piglets were significantly lower than those in the control group after the challenge [17]. Furthermore, *E. faecalis* AG5, a probiotic strain, alleviated obesity in mice, and TNF-α expression was decreased in liver samples [29]. Previous studies have indicated that whey fermented by *E. faecalis* M157 significantly inhibited IL-1β induced by the lipopolysaccharide of *Porphyromonas gingivalis* in RAW 264.7 cells [30]. Notably, our study found that dietary supplementation with *E. faecalis* HHP003 in cats with mild diarrhea significantly reduced the serum levels of pro-inflammatory cytokines TNF-α and IL-1β.

The intestine influences and modulates the host’s inflammatory status by regulating homeostatic balance, inducing immune tolerance, and preventing pathological immune responses. As a 36 kDa protein of the S100 family, calprotectin is mainly derived from neutrophil granules. Notably, its levels serve as a reliable indicator of inflammation severity, increasing in parallel with the extent of inflammatory activity [31]. Importantly, this study revealed that blending the probiotic strain *E. faecalis* HHP003 into the diet markedly decreased the fecal calprotectin concentrations in the EF group relative to the MD group. Consistent with the findings of previous studies, supplementation with multi-strain probiotics could markedly decrease the fecal calprotectin levels in healthy domestic shorthair cats [32]. More interestingly, multiple probiotics have been applied to control gut dysbiosis and inflammatory responses, and the fecal calprotectin levels were significantly decreased in mice with induced atopic dermatitis following probiotic administration [33]. LPS is a specific component of the cell wall of Gram-negative bacteria and can stimulate host immune cells to produce inflammatory cytokines [34]. When the intestinal barrier is damaged, LPS in the intestinal lumen can enter the peripheral circulatory system, triggering or exacerbating systemic inflammatory responses [35]. In this study, cats in the EF group exhibited a significantly lower LPS level relative to the MD group. These findings suggest that *E. faecalis* HHP003 may preserve intestinal barrier integrity and support gastrointestinal health in felines.

The gut microbial profile of cats is closely linked to several gastrointestinal disorders, including chronic enteritis and diarrhea [4]. Notably, probiotics have been used as a nutritional strategy to promote health and modulate the gut microbiota in both humans and animals [36]. Previous investigations have established that Bacillota and Bacteroidota represent the predominant phyla within the feline gut microbial community [6]. A study investigating the effects of *Bacillus licheniformis*-fermented products as probiotics on the gut microbiota and clinical manifestations in cats with chronic diarrhea found that probiotic supplementation correlated with increased Bacillota and Bacteroidota in cats experiencing diarrhea [37]. The present study found that the Bacillota level in cats of the EF group was significantly higher than those in the CON and MD groups after 42 days’ intervention. Although the Bacteroidota level in the CON group was significantly higher than that in the MD and EF groups, the Bacteroidota level in the EF group was increased compared with the MD group. Supporting our results, the abundance of Lachnospiraceae and Ruminococcaceae was increased in dogs with intestinal inflammation after probiotic supplementation [38]. As a core component of the gut microbiota, Lachnospiraceae colonizes the gut from birth, accumulates throughout the host’s lifespan, and can utilize dietary polysaccharides such as arabinoxylan, inulin, and starch to produce short-chain fatty acids (SCFAs) [39,40]. Certain species within Ruminococcaceae can break down complex carbohydrates (e.g., cellulose), and SCFAs produced not only provide energy for intestinal epithelial cells but also regulate the intestinal immune system and reduce inflammatory responses [41]. Moreover, as a genus belonging to the family Lachnospiraceae, *Blautia* has attracted considerable attention since its establishment due to its contributions to alleviating inflammatory and metabolic diseases, as well as its antimicrobial activity against specific microorganisms [39]. Consistent with our results, 16S rRNA gene amplicon sequencing demonstrated that mice administered *E. faecalis* for 14 days showed increased relative abundances of Ruminococcaceae and *Blautia* in the intestinal microbiota [42]. More interestingly, *Escherichia-Shigella* represents a typical harmful bacterium with the ability to disrupt the intestinal structure [43]. In the present study, supplementation with *E. faecalis* HHP003 in cats with mild diarrhea reduced the abundance of *Escherichia-Shigella* compared with the CON and MD groups, thereby contributing to the alleviation of intestinal disorders in the cats. Previous studies have found that supplementation with *E. faecalis* in avian pathogenic *Escherichia coli*-challenged broilers decreased the abundance of *Escherichia-Shigella*, which may help attenuate intestinal disruptions in broilers [44]. In brief, our results found that *E. faecalis* HHP003 regulated the composition of intestinal microorganisms in cats with mild diarrhea. Interestingly, despite supplementation with *E. faecalis* HHP003, the relative abundance of *Enterococcus* in the EF group was lower than that in the CON and MD groups. This might possibly be due to the inability of 16S rRNA sequencing to distinguish *E. faecalis* from other enterococci [45], as well as the ability of probiotic *Enterococcus* strains to potentially suppress other *Enterococcus* species through bacteriocin production [46]. We also recognize that the lack of baseline microbiota data limits our interpretation; therefore, future studies incorporating baseline sampling and absolute quantification are necessary to validate this finding.

As intermediate and final products of metabolic pathways, metabolites play important roles in the overall health and metabolic processes of organisms [47]. KEGG pathway topology analysis revealed significant differential metabolic pathways among the CON, MD, and EF groups. Among them, arachidonic acid metabolism and steroid hormone biosynthesis were common significantly differential pathways across all pairwise comparisons between groups. Arachidonic acid metabolism is a core pathway regulating intestinal inflammatory responses, and its metabolites serve as key mediators of pro-inflammatory and anti-inflammatory signals [48]. Meanwhile, steroid hormone biosynthesis is closely associated with immune regulation and intestinal mucosal repair [49]. Notably, *E. faecalis* HHP003 may reverse the lipid and steroid hormone metabolic disorders induced by mild diarrhea, thereby alleviating intestinal inflammation and maintaining mucosal homeostasis. This finding is consistent with the results of serum inflammatory factors: the levels of TNF-α and IL-1β were significantly lower in the EF group than those in the MD group. In addition, comparison of the EF and CON groups also revealed differential pathways such as α-linolenic acid metabolism and glutathione metabolism. Glutathione metabolism, the major antioxidant defense pathway in the body [50], exhibited changes in the EF group, indicating that *E. faecalis* HHP003 may alleviate oxidative stress induced by mild diarrhea by regulating the antioxidant capacity of cats. As an important polyunsaturated fatty acid metabolic pathway, α-linolenic acid metabolism further supplements the regulatory effect of *E. faecalis* HHP003 on lipid metabolism and anti-inflammatory responses [51]. Comparison between the EF and MD groups also revealed significant differences in primary bile acid biosynthesis, a pathway closely related to the gut microbiota structure and nutrient absorption [52], suggesting that *E. faecalis* HHP003 may regulate bile acid metabolism by reshaping the gut microbiota, thereby improving intestinal nutrient utilization and barrier function. This is corroborated by the fecal calprotectin results, which indicate decreased intestinal inflammation in the EF group. However, the conclusions drawn from the KEGG pathway analysis based on untargeted metabolomics in this study remain preliminary and require further validation. Future studies may employ other omics approaches in combination to elucidate the precise regulatory mechanisms underlying these pathways.

Assessment of Spearman correlations linking serum differentially abundant metabolites with genus-level gut microbial composition further established the functional association between microbial alterations induced by *E. faecalis* HHP003 and systemic metabolic changes. 4-Hydroxyproline, a characteristic amino acid of collagen and elastin, serves as a key biomarker of intestinal mucosal integrity, collagen metabolism, and mucosal repair [53]. As typical symbiotic bacteria in the feline intestine, *Bifidobacterium* have been reported to be associated with intestinal anti-inflammatory effects and homeostasis [54]. This positive correlation closely links the gut microbiota, amino acid metabolism, and intestinal mucosal health. In addition, tryptophan-related metabolites (5-Hydroxy-L-Tryptophan and N-Formyltryptophan) were significantly correlated with multiple core microbial genera. N-Formyltryptophan correlated positively with beneficial bacteria and negatively with opportunistic pathogens, suggesting its potential as a metabolic marker for intestinal health. The regulation of N-Formyltryptophan by *E. faecalis* HHP003 may represent an important mechanism for inhibiting pathogenic bacteria and enriching beneficial flora. Meanwhile, 5-Hydroxy-L-Tryptophan, a key intermediate in serotonin synthesis, was negatively correlated with the genus *Blautia*. Serotonin is a crucial neurotransmitter regulating intestinal peristalsis and barrier function, and its metabolic disorder is closely associated with diarrhea and intestinal dysfunction [55]. Therefore, *E. faecalis* HHP003 may regulate the tryptophan–serotonin metabolic axis via *Blautia*, thereby modulating intestinal peristalsis and inflammatory status. Although cats with a history of parasitic infections or clinical signs of giardiasis were excluded, specific PCR testing or coproparasitoscopic examination for Giardia infection was not performed. Future studies should include active screening for Giardia to completely rule out potential interactions with probiotic supplementation.

## 5. Conclusions

In summary, compared with the MD group, cats in the EF group showed significant improvements in serum inflammatory response, intestinal inflammation and barrier markers, gut microbiota structure, and metabolic homeostasis. These results underscore the desirable characteristics of *E. faecalis* HHP003 in improving intestinal health in cats, indicating that this strain has the potential to be applied in pet food to enhance the health status of cats.

## Figures and Tables

**Table 1:** The basal diet composition and nutrient levels.

Diet Composition	%	Nutrient Content	%
Chicken meal	54.50	Crude protein	41.65
Potato starch	19.00	Crude fat	20.28
Chicken fat	8.00	Ash	7.87
Chicken liver powder	5.00	Moisture	7.12
Rice	4.00	Crude fiber	1.82
Alfalfa meal	3.00		
Tapioca	3.00		
Fish oil	2.00		
Salt	0.50		
Mineral complexes and vitamins ^1^	0.50		
Choline chloride	0.30		
Taurine	0.20		

^1^ The vitamin and mineral complex content per kilogram of feed is as follows: vitamin E (156 IU), vitamin D_3_ (1000 IU), vitamin B_12_ (0.20 mg), vitamin B_6_ (13.0 mg), vitamin B_5_ (88.0 mg), vitamin B_3_ (120 mg), vitamin B_2_ (30.0 mg), vitamin B_1_ (32.0 mg), and vitamin A (14,500 IU); Mn (MnSO_4_) 20.0 mg, Zn (ZnSO_4_) 68.0 mg, Cu (CuSO_4_) 7.00 mg, Fe (FeSO_4_) 100 mg, Co (CoSO_4_) 1.00 mg, Se (Na_2_SeO_3_) 0.50 mg, and I (CaI_2_) 20.0 mg.

## Data Availability

The data supporting the findings in this research are available by contacting the corresponding author on request.

## Appendix A

**Table A1:** Fecal condition score.

Score	Standard
1	Extremely dry and crumbly
1.5	Dry and hard
2	Well-formed; no residue when picked up
2.5	Well-formed; slightly moist surface; slightly sticky when picked up
3	Begins to lose shape; increased moisture on surface; clear residue when picked up
3.5	Significantly moist; retains some shape
4	Mostly unformed; loose and sticky
4.5	Diarrheic; minimal formation
5	Severe diarrhea

**Table A2:** ELISA kit detection information.

Detection Indexes	Kit Catalog Numbers	Detection Samples	Detection Range
TNF-α	MM-67524O2	serum samples	5–185 ng/L
IL-1β	MM-2552O2	serum samples	2–90 ng/L
IL-6	MM-67519O2	serum samples	6–280 ng/L
calprotectin	MM-67647O2	fecal samples	10–450 pg/mL
CRP	MM-67620O2	fecal samples	60–2200 μg/L
LPS	MM-67540O2	serum samples	5–220 ng/L
zonulin	MM-67664O2	serum samples	10–700 ng/L
